# Factors affecting superovulation induction in goats (*Capra hericus*): An analysis of various approaches

**DOI:** 10.3389/fvets.2023.1152103

**Published:** 2023-03-24

**Authors:** Samiullah Khan, Muhammad Ameen Jamal, Ibrar Muhammad Khan, Irfan Ullah, Abdul Jabbar, Nazir Muhammad Khan, Yong Liu

**Affiliations:** ^1^Anhui Province Key Laboratory of Embryo Development and Reproduction Regulation, Anhui Province Key Laboratory of Environmental Hormone and Reproduction, School of Biological and Food Engineering, Fuyang Normal University, Fuyang, China; ^2^The Scientific Observing and Experimental Station of Crop Pest in Guiyang, Ministry of Agriculture, Institute of Entomology, Guizhou University, Guiyang, China; ^3^Kunming Institute of Zoology, Chinese Academy of Sciences, Kunming, China; ^4^College of Biotechnology, Jiangsu University of Science and Technology, Zhenjiang, Jiangsu, China; ^5^Faculty of Veterinary and Animal Sciences, University of Poonch, Rawalakot, Pakistan; ^6^Department of Zoology, University of Science and Technology, Bannu, Pakistan

**Keywords:** strategies, factors, superovulation, hormonal protocols, goat

## Abstract

Goats are generally called a “poor man's cow” because they not only provide meat and milk but also other assistance to their owners, including skins for leather production and their waste, which can be used as compost for fertilizer. Multiple ovulation and embryo transfer (MOET) is an important process in embryo biotechnology, as it increases the contribution of superior female goats to breeding operations. The field of assisted reproductive biotechnologies has seen notable progress. However, unlike in cattle, the standard use of superovulation and other reproductive biotechnologies has not been widely implemented for goats. Multiple intrinsic and extrinsic factors can alter the superovulatory response, significantly restricting the practicability of MOET technology. The use of techniques to induce superovulation is a crucial step in embryo transfer (ET), as it accelerates the propagation of animals with superior genetics for desirable traits. Furthermore, the conventional superovulation techniques based on numerous injections are not appropriate for animals and are labor-intensive as well as expensive. Different approaches and alternatives have been applied to obtain the maximum ovarian response, including immunization against inhibin and the day-0 protocol for the synchronization of the first follicular wave. While there are several studies available in the literature on superovulation in cattle, research on simplified superovulation in goats is limited; only a few studies have been conducted on this topic. This review describes the various treatments with gonadotropin that are used for inducing superovulation in various dairy goat breeds worldwide. The outcomes of these treatments, in terms of ovulation rate and recovery of transferrable embryos, are also discussed. Furthermore, this review also covers the recovery of oocytes through repeated superovulation from the same female goat that is used for somatic cell nuclear transfer (SCNT).

## 1. Introduction

Goat production plays an important role in the livelihoods of farmers, especially in developing countries. In the last 20 years, the world's population of goats has increased by 49%, whereas sheep and cattle have shown a slow increase of 15 and 14%, respectively (1). Goats provide various animal by-products such as meat, milk, and hides to poor families; they are also a viable option for commercial farming, making a valuable contribution to the livestock industry (2–4).

The application of multiple ovulation and embryo transfer (MOET) plays a vital role in the global trade of genetic resources, conserving endangered species, minimizing the risk of exotic diseases and the cost of production, and eliminating transportation-related stress (5, 6). This technology made great progress in the 1970s, largely due to its remarkable practice in cattle (4). The sequence of events leading to the ET usually starts with superovulation. Superovulation is an important phase in the MOET program, as it has the potential to increase the number of genetically superior donors, especially when there is a high demand for such animals (7, 8). Under normal circumstances, a goat typically ovulates 1–3 eggs per estrus cycle. However, by administering approximate doses of FSH hormone through superovulation, approximately 10–20 available oocytes with 13 transferrable embryos (8) can be obtained (4). The fundamental idea behind superovulation involves the artificial administration of exogenous gonadotropins, such as FSH or PMSG, to promote the development of a greater number of follicles, ultimately leading to ovulation (4).

However, the widespread implementation of MOET in goats has been hindered due to the high cost of hormones (9) and the unpredictable response of donor animals to superovulation hormones. Ovarian responses in goats can vary and are affected by a number of factors such as breed, age, nutrition, animal management practices and selection, stress, gonadotrophin supply, and seasonal cyclic activity (8, 10–21). These factors could negatively influence superovulation outcomes by reducing the quality of oocytes, which ultimately decreases the number of transferrable embryos for ET. In addition, there are some other disadvantages associated with the use of multiple injections (6–10 injections over 3–5 days), including the time and labor investments required (22).

A robust superovulation approach that meets the requirements of both researchers and producers in terms of ovarian response predictability and dependability has yet to be created. For instance, a simplified superovulatory protocol that involves fewer FSH injections (i.e., single or only two injections) while maintaining the same level of ovarian response in terms of recovered and transferrable embryos would be ideal (21). Further, these simplified protocols will also facilitate easier implementation for a large herd of animals while requiring less time and resources (18–21).

This review examined recent studies on various hormonal protocols for inducing superovulation in goats, including attempts to simplify these protocols, and discusses the outcomes of the approaches as well as the various factors that can affect superovulation. Additionally, new approaches applied to superovulation in goats are explored.

## 2. Hormonal protocols and their outcomes

Superovulation can be performed mostly using exogenous gonadotrophins such as PMSG and FSH. However, other rarely used hormones are horse anterior pituitary (HAP) extract, human chorionic gonadotrophin (hCG), or gonadotrophin-releasing hormone (GnRH) (17). PMSG is a unique member of the gonadotropin family, also known as equine chorionic gonadotropin (eCG), which is a complex glycoprotein containing both FSH and LH-like activities (23). This hormone is fairly valuable for inducing superovulation in goats (24, 25). This hormone has an advantage over FSH because it can easily be applied to an open flock in a single subcutaneous or intramuscular injection given 1 or 2 days prior to the last synchronization treatment at a dose of 750–2,000 IU. Owing to its simplicity, a single injection not only causes less stress by minimizing excessive handling but is also cost-effective in comparison to FSH multiple injection protocols (24, 26, 27). However, PMSG_influenced the steroidal hormone pattern, leading to the premature regression of the corpus luteum (28) and an increase in the number of persistent large follicles that eventually failed to ovulate, resulting in reduced ovarian response (27, 29). It also causes follicular steroid secretion, which can interfere with sperm and gamete transport, oocyte maturation, and early preimplantation embryo development, thereby altering the endogenous endocrine environment (30). Due to its long half-life (72 h), PMSG injection can cause a high incidence of anovulatory follicles and early degeneration of the corpus luteum (CL) in goats' ovaries.

FSH is secreted from an anterior lobe of the pituitary, causing the growth and development of small follicles on the ovaries. This hormone is commonly injected multiple times over a period of 3 to 4 days during the follicular phase and 48 h before the sponges are removed. However, as discussed earlier, multiple injections are time-consuming, laborious, and stressful for animals (31, 32), which ultimately decreases the superovulatory response (33).

Comparative studies have shown that FSH is superior to PMSG and produces more transferable embryos (5, 19, 34). In this study, FSH therapy also produced more embryos than eCG therapy, and embryos were recovered from indigenous dairy goat breeds such as *Jamunapari, Angora, Jakhrana, Tellicherry*, and crossbred (*Boer Katjang*) goats (17, 19, 34–38) (Table 1).

**Table 1:** Comparison of FSH and PMSG for the induction of the superovulatory response.

**Hormone**	**Dose**	**Mode of injections (d)^1^**	**Estrus signs (hours)**	**Ovulation rate**	**Embryos recovered**	**Transferable embryos**	**References**
PMSG	1,000 IU	Single	17^a^	5.17^a^	3.33^a^	_	(38)
FSH	Multiple doses	6^d^	26^b^	12.07^b^	8.00^b^	_	
ECG	1,500 IU	Single	36^a^	6.73^a^	0.53^a^	_	(17)
FSH	200 mg	6^d^	27^a^	6.40^a^	2.00	_	
PMSG	1,200 IU	Single	25.8^a^	3.9^a^	2.3^a^	_	(34)
FSH	21 mg	8^d^	29.0^a^	12.3^b^	9.3^b^	_	
PMSG +hCG	750 IU	Single	38.4^a^	8.4^a^	5.8^b^	3.8^c^	(19)
FSH +hCG	12.5 IU	8^d^	36^a^	11.8^a^	8.0^b^	5.2^c^	
FSH^2^+hCG	25 units	Three doses	42^a^	11.6^a^	6.6^b^	5.4^c^	
PMSG	750 IU	Single	33.3^a^	12.5^c^	0.8^c^	0.1^c^	(37)
FSH	4,3,2,1 mg	Twice daily, 4 days	32.0^a^	14.7^c^	7.0^d^	6.7^d^	
PMSG	500–750 IU	Single	_	11.70^a^	2.50^a^	_	(36)
FSH	16 mg	8^d^	_	16.55^b^	4.72^b^	_	
PMSG	750 IU	Single	_	3.00	3.00	3.00	(35)
FSH	16 mg	6^d^	_	8.80	7.00	5.11	
FSH	3 mg^2^, 12 mg	6^d^	_	7.33	5.66	3.33	
PMSG FSH	750 IU and 12 mg	6^d^	_	7.25	2.26	0.75	

^1^Decreasing dose.

^2^The animals were primed with 3 mg of FSH-P on day 4 of the sponge insertion. A total of 12 mg of FSH was administered in six injections 48 h before sponge removal.

Values with different superscripts a–d within the column show a significant difference at *p* < 0.05.

Sustained exposure to low levels of FSH hormone in multiple doses is required to achieve superovulation (39). Few successful attempts have been made to simplify these protocols by combining FSH with a low dose of PMSG in a single injection (40) or by using a single injection of porcine FSH (41). In Canindé goats, the mean number of cumulus-oocyte complexes (COCs) recovered did not show any significant difference between the treatments. The number of COCs retrieved was 10.8 oocytes for a single injection of 70 mg Folltropin-V plus 200 IU eCG and 11.7 oocytes for five doses of 120 mg (42).

In contrast, Lehloenya (43) found that using a simplified superovulation treatment with FSH and PMSG was less effective than the traditional protocol based on multiple FSH injections. The lower response to the simplified superovulation treatment could be due to an increase in the number of large follicles during a single injection of FSH and PMSG (44). These contradictory results suggest that the ovarian response may depend on the type of FSH preparation used and the appropriate dosage of FSH and PMSG.

Many studies obtained satisfactory ovarian responses using simplified superovulatory techniques in cattle (45–49). Many research groups also obtained a similar ovarian response in sheep when simplified protocols were compared to multiple injection protocols (32, 39, 41, 50–56). The studies conducted on goats concerning the subsequent stage of embryo yield and quality are limited, suggesting the need for further research, particularly on large-scale embryonic production in the context of the superovulation protocol followed by AI (55, 57). Moreover, the endocrinological bases of simplified protocols are not well understood, leaving considerable room for studying their relationship with the endocrine profile. Such research could potentially reduce the labor and hormone costs associated with large-scale production.

Human chorionic gonadotrophin (hCG) or gonadotrophin-releasing hormone (GnRH) is rarely used in goats, as well as in sheep and cattle. Treating crossbred goats with hCG yielded a higher number of CL and recovered oocytes (10.9 and 3.10, respectively) compared to goats treated with GnRH (1.90 and 0.7, respectively) after supplementing hCG and GnRH with eCG at the rate of 1,500 IU. However, despite the higher number of recovered oocytes, there was no difference in the average number of embryonic recoveries, which made it difficult to enhance embryonic production (58).

## 3. Factors affecting the superovulatory response

Various factors limit the practicability of MOET for goats and other domestic animals. Therefore, it is important to manipulate these factors to improve the ovarian response. Major factors are discussed in this section, including several extrinsic factors (e.g., sources, the purity of gonadotrophins, and their administration) and intrinsic factors (e.g., breed, age, nutrition, and season).

**Figure 1:**
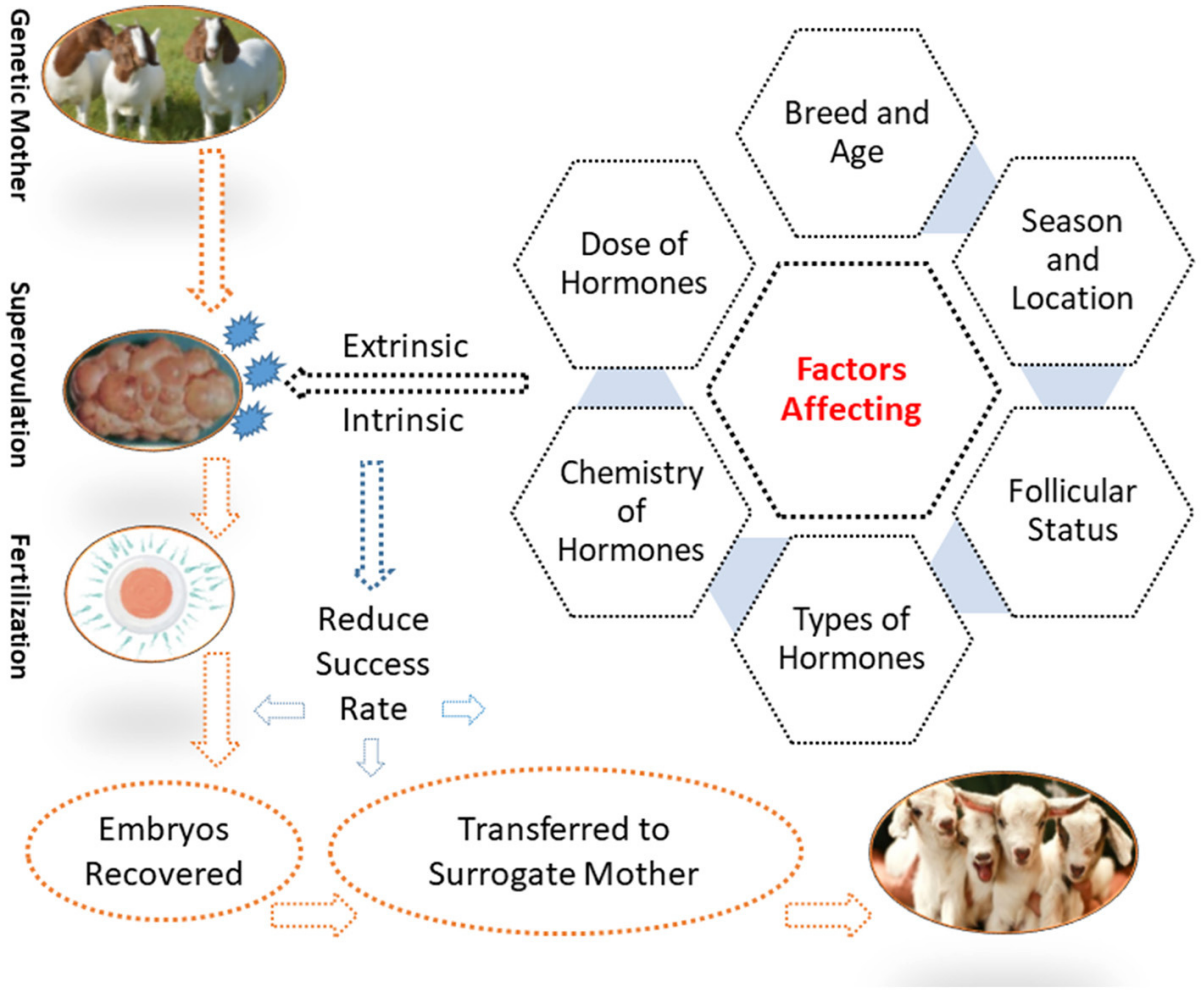
Factors affecting the superovulatory response in goats.

The factors that contribute to the variability in the ovarian response of goats are shown in Figure 1.

### 3.1. Types of hormones and the number of injections

FSH was found to produce higher ovulation rates and transferrable embryos compared to other PMSGs and eCG. The type of hormones and the number of injections may contributed to the superovulatory response. Similar observations for FSH and PMSG were also made in ewes (24, 55, 59, 60). Hormonal protocols and their outcomes are discussed in detail in Table 1.

### 3.2. Age of the animals

With varying ovarian responses to hormonal therapies, age may also be regarded as a limiting factor (6, 10). Mahmood et al. (36) reported a higher number of CL and recovered embryos (RE) (19.10 and 5.40) for the 4- to 6-year age group than (13.25 and 4.00) for the 1.5- to 3-year age group. A higher response was also obtained for 3–4-year-old goats than for 1–2-year-old goats (61). Similarly, Chang et al. (10) injected six or seven injections of FSH, given in decreasing doses over a four-day period, to goats of different ages. The results revealed a higher ovarian response, indicated by better ovulation rates, among goats aged 12–23 months compared to those aged 7–12 months. Additionally, a better proportion of transferable embryos was observed in the 1–5-year-old group than in the 0.7–1.0 year age group. In sheep, it was found that older ewes (24–60 months) produced a greater number of recovered and transferrable embryos compared to the younger group (8–12 months) when given a superovulation treatment consisting of eight decreasing doses of 160–200 mg (16).

The higher ovarian response observed in older goats could be due to a fully functional ovary, prior pregnancy experience (1–2 pregnancies), and a large body frame (10, 16). In sheep, a study reported a higher ovulation rate among 1–2-year-old ewes compared to 3–4-year-old ewes. This may be due to young animals experiencing fewer disease incidents and health problems (62).

### 3.3. Dosage of the hormones

The effectiveness of the FSH dose was also investigated in relation to an ovarian response (Table 2). Sánchez-Davila et al. (9) reported that administering 145 and 215 mg of FSH in seven doses over 3.5 days produced similar ovarian responses, but significantly higher responses were observed when the dose was reduced to 80 mg using the same protocol. Abdullah et al. (15) found no significant difference in the number of recovered and transferrable embryos while injecting FSH doses of 8.8 mg and 14.08 mg, starting 48 h before CIDR removal. Similarly, Rahman et al. (17) did not find any significant difference in the ovulation rate and transferrable embryos while injecting 5 and 3 mg of FSH/kg body weight, but they did observe a decreased ovarian response while using a higher dose of 8 mg/kg body weight. In sheep, a few studies (55, 60, 63) found no difference with a slight reduction in the FSH dose. These results suggest that a small reduction in dosage may not have a significant impact on ovarian response. However, a large reduction may lead to a decreased ovarian response. Therefore, an optimal reduction in hormonal dose can reduce the cost of hormones without negatively affecting the ovarian response, especially in large-scale *in vivo* embryonic production.

**Table 2:** Effect of different hormone doses on the superovulatory response.

**Hormone**	**Frequency**	**Dose rate**	**Oestrous onset (hours)^1^**	**CL^2^**	**Recovered embryos**	**Transferrable embryos**	**Reference**
FSH	Six (dd)^3^	3 mg/kg bd.wt^4^	_	9.20^a^	3.0^a^	2.8^b^	(17)
FSH	Six (dd)^3^	5 mg/kg bd.wt^4^	_	10.5^a^	7.0^b^	6.71^b^	
FSH	Six (dd)^3^	8 mg/kg bd.wt^4^	_	7.38^a^	2.0^a^	1.50^a^	
FSH	Seven (dd)^3^	80 mg	28.8	4.0^b^	1.0^b^	0.8^b^	(9)
FSH	Seven (dd)^3^	145 mg	24.4	13.4^a^	3.4^a^	3.2^a^	
FSH	Seven (dd)^3^	215 mg	26.4	11.6^a^	5.7^a^	5.6^a^	
FSH	Multiple doses	8.8 mg	_	11.6^b^	3.2^ab^	2.4^a^	(15)
FSH	Multiple doses	14.08 mg	_	16.9^b^	5.4^b^	2.0^a^	

^1^Interval from sponge removal to the estrous onset (hours).

^2^No of corpura lutea.

^3^Decreasing dose.

^4^Body weight.

Values with different superscripts a and b within the column show a significant difference at *p* < 0.05.

### 3.4. Breed

The ovarian response of the breed to superovulatory hormones was found to be uneven and inconsistent. Nuti et al. (64) found that, when administering a total of 15 mg of FSH through six injections, a higher percentage of ovarian response was observed in *Nubian* female goats compared to *Alpine* goats. Conversely, Kiessling et al. (65) found no breed effect on ovarian response in *Saanen, Alpine, Nubian*, and *La Mancha* goats when administering superovulation. However, they did not observe limited differences in ovarian response between the *Boer* and indigenous breeds when using pFSH 200 mg/dose injected in seven decreasing doses. The differences were noted in terms of total CL (12.78 and 14.37), total embryos (8.40 and 8.12), and transferable embryos (6.60 and 6.00) (66).

The administration of different hormones during the superovulation process led to diverse ovarian responses in different breeds of sheep. A significantly higher number of CL and embryos (13.7 and 7.9) were observed for the *Rubia del Molar* breed compared to the *Negra de Colmenar* (10 and 4.3) and *Manchega* ewes (9.8 and 6.7) after administering the same eight decreasing doses of oFSH for superovulation (13). Furthermore, a higher number of CL and transferable embryos were observed in *Chios* than in *Friesian* breeds when subjected to multiple doses of pFSH (13, 67). Conversely, there was no difference in ovarian response between *Corriedale* and *Bond* donor ewes when injected with a split-single dose of 180 mg of FSH dissolved in 10 mg/ml 750 kDa hyaluronan (56). These results indicated that variation in ovarian response among different breeds subjected to the same hormonal protocol may be due to the varying kinetic behavior of the exogenous gonadotrophin, follicular status, and function, or environmental influences (S.7).

### 3.5. Season and location

After superovulation protocols, the ovarian response during different seasons was found to be inconsistent (Table 3). Specifically, a significantly higher ovulation rate (28.7) and number of recovered embryos (15.1) were obtained from *Nubian* goats during the early breeding season compared to the ovulation rate (9.30) and the number of recovered embryos (3.3) during the late breeding season (68). However, Chang et al. (10) obtained a better ovarian response in breeding (17.24) than in non-breeding (10.40) and late breeding (14.32) seasons in Shandong province, China.

**Table 3:** Different seasons influence the ovarian response.

**Season**	**Estrus**	**Ovulation rate**	**Embryos recovered**	**Transferred embryos**	**References**
Breeding	24.9^a^	17.5	16.4	12.3	(8)
Non-breeding	30.5^b^	21.3	16.5	13.1	
Non-breeding	_	10.40^a^	_	0.82^1ab^	(10)
Breeding	_	17.24^b^	_	0.85^1a^	
Late breeding	_	14.32^ab^	_	0.66^1b^	
Breeding	_	7.98	2.93^a^	1.64^a^	(22)
Non-breeding	_	8.95	7.16^b^	3.96^c^	
Breeding	_	9.74	4.74	2.34	
Non-breeding	_	10.03	6.47	3.03	
Early breeding	_	28.7	15.1	_	(68)
Late breeding	_	9.3	3.3	_	

^1^Represent the proportion of transferrable embryos.

Values with different superscripts a–c within the column show a significant difference at *p* < 0.05.

Superovulatory treatment and season did not affect the ovulation rate (7.98 vs. 8.95). However, the number of recovered (2.93 vs.7.16) and transferrable (1.64 vs. 3.96 embryos) embryos were higher in the non-breeding season than in the breeding season, respectively (22). There was no significant difference between the ovulation rate (10.2 vs. 9.22) and the total number of embryos (4.10 vs. 5.44) during the breeding season when we administered FSH in seven decreasing doses and three FSH plus one PMSG injection group, respectively. Similarly, the ovulation rate (10.74 and 9.32) and the total number of embryos (5.95 and 7.00) were also similar between these two groups during the non-breeding season when the same superovulatory protocol was used (22). Additionally, there was no significant difference between the breeding and non-breeding seasons when we treated goats using these two protocols. Similarly, similar numbers of recovered embryos (16.4 and 16.5) were obtained when superovulation with multiple dosages of FSH was carried out during the breeding and non-breeding seasons, respectively (8). Baril and Vallet (69) found no difference in ovarian response when inducing superovulation in *Alpine* goats with porcine FSH during and out of the breeding season. The lack of differences in the number of ovarian structures recovered and transferable embryos between seasons recorded per donor may be due to the similarity of climatic conditions during both seasons (9). It is evident that season has an effect on the time to onset and the duration of the induced estrous period following superovulation in goats, which is important to consider, especially for fixed-time AI (8).

## 4. Approaches to superovulation

### 4.1. Inhibin immunization and conventional superovulatory protocols

Inhibin, a member of the transforming growth factor beta (TGF-β) superfamily, is a gonadal hormone that has a negative effect on the secretion of FSH from the gonadotropic cells of the pituitary gland (70). The use of immunization against inhibin, along with conventional superovulation, may be considered a viable alternative to achieve maximum output in goats (71). The use of inhibin antiserum in treated goats resulted in a higher ovarian response compared to the control group, with a greater number of follicles (13.5 vs. 5.3) and ovulation rate (4.2 vs. 1.8) (72). There was approximately a 4-fold increase in the ovulation rate (7.6) in goats actively immunized against inhibin compared to the control (1.7) group. The onset of estrus was also shorter (46.8 h) for the inhibin-immunized group than the control group (54.4 h) (73). Holtz et al. (74) reported obtaining 5.5 transferrable embryos using a combination of inhibin immunization and conventional superovulation protocol, which is lower than the 13.1 embryos obtained by Lehloenya et al. (8) using conventional superovulation alone. Studies have shown that the use of immunization against inhibin results in significantly lower ovulation rates and fewer embryos recovered compared to conventional FSH protocols. This finding is consistent with previous studies conducted by Pendleton et al. (37), Lehloenya et al. (8), Palanisamy et al. (38), and a few other studies (75, 76). However, in ewes that were both treated with FSH and actively immunized against porcine inhibin α-subunit, a higher ovulation rate of 12.1 was observed. In contrast, ewes that were treated with the conventional FSH superovulation protocol alone had an ovulation rate of 5.0 (77). In cattle, using inhibin immunization in combination with a superovulation protocol resulted in a higher embryo yield and ovarian response compared to conventional protocols (78, 79). In mice, the use of inhibin antiserum in combination with conventional superovulatory protocols has recently resulted in a one-third increase in the number of oocytes compared to the control group (80). Inhibin immunization, along with conventional or simplified superovulatory methods, has not been broadly studied in goats. Further studies are required to study its effect on the ovulation rate, *in vitro* fertilization among different breeds, and the further processing of oocytes to observe their quality and quantity.

### 4.2. Super-stimulation of the first follicular wave: The day-0 protocol

Recent studies on follicular dynamics have enabled researchers to develop new superovulation protocols for embryonic production, such as the day-0 protocol. The day-0 protocol comprised the synchronization of ovulation and the emergence of the first wave. The day-0 protocol involved inserting CIDR for 5 days, followed by an injection of (PG) F2α to induce luteolysis. To synchronize the ovulation procedure, a dose of approximately 200–300 IU of eCG was injected upon CIDR withdrawal. Additionally, a single dose of GnRH was administered 36 h after CIDR withdrawal to ensure ovulation. Day 0 was estimated as 84 h after CIDR withdrawal (i.e., soon after ovulation). The FSH injections were given two times/day with decreasing doses starting 84 h after CIDR removal, and two half-doses of PGF2a were administered with the 5th and 6th FSH treatments. To synchronize the LH peak and ovulation, the GnRH analog was administered 24 h after the first PGF2a treatment. Timed AI was performed using laparoscopy under moderate sedation with frozen-tawed semen at 16 and 26 h after GnRH administration (14, 81).

In goats, wave-like patterns occur during follicular dynamics (82). The most frequent finding is the occurrence of four follicular waves during an estrous cycle in goats, but high variability among cycles has also been reported. Each follicular wave is preceded by a transient increase in the concentration of FSH. Generally, one to three follicles grow to a diameter of 5 mm after the development of the waves. However, the remaining (medium, 4–5 mm; small, 2–4 mm) enter the atresia. Further aspects of follicular wave patterns in goats have been reviewed by Menchaca et al. (14) and Rubianes and Menchaca (83).

To synchronize ovulation and the emergence of the first follicular wave, Menchaca et al. (81) compared the day-0 protocol with the traditional multiple FSH protocol with six decreasing doses. During the breeding season, the yield of transferable embryos increased from 2.6 in goats treated with the conventional superovulatory protocol to 4.9 in goats treated with the day-0 procedure.

Similarly, during the non-breeding season, the use of the conventional protocol resulted in an ovulation rate of 10.7, embryos recovered at a rate of 7.6, and a yield of transferrable embryos (4.2). In comparison, the day-0 protocol increased the ovulation rate to 14.3, and the number of embryos recovered remained the same at 7.6. However, the yield of transferrable embryos increased to 5.9. The day-0 protocol also yielded a high ovarian response compared to the traditional protocol. However, as there are few studies on day 0 of the superovulatory protocols, further research is warranted to improve embryonic production efficacy.

In goats, the presence of a dominant follicle had a deleterious effect on follicle recruitment and the superovulatory response. However, using the day-0 protocol resulted in a higher percentage of females responding, an increased number of CL, and a higher number of grade 1 and 2 embryos during both breeding and non-breeding seasons in comparison to the traditional protocol. Large follicles at the beginning of the superovulation treatment are associated with the total number of unfertilized ova, while medium follicles (4–5 or 6 mm) at the beginning of the superovulation treatment are directly associated with the number of recovered and viable embryos and transferable embryos (66, 84). The ovarian response in terms of the number of corpora lutea (15.3) was positively correlated to the total number of follicles with a diameter of 2–6 mm at the beginning of the FSH treatment. A high number of larger follicles (≥7 mm) had a negative effect on the ovulation rate (84). The modified (day 0) protocol should be initiated when there is a suitable number of follicles from the second category present on the ovaries (85).

### 4.3. Repeated superovulation

The administration of hormonal treatment at intervals to recover oocytes repeatedly from the same animal is known as “repeated superovulation.” A study conducted by Lehloenya et al. (86) reported a significant reduction in the number of embryos recovered (11.7 vs. 3.8) and the number of transferrable embryos (10.7 vs. 3.8) during the first and repeated superovulation in the natural breeding season; however, the ovulation rate was not affected (14.8 and 16.8). Chang et al. (10) did not observe any reduction in the number of oocytes recovered during the first and second oocyte cycles of superovulation, but significant decreases were observed during the third superovulation cycle. This decrease in ovarian response may be attributed to genital tract adhesion after repeated flushing (10); thus, limited potential for surgically obtaining repeated embryo collections from the same animal is demonstrated (16, 87).

These findings suggested that the decrease in ovarian response during repeated superovulation could be due to the formation of post-operative adhesions in the reproductive tract, which may have an adverse effect on the ovarian function or uterine cells, resulting in a reduced number of embryos recovered during successive treatments (16, 32). It is evident that repeated use of animals for superovulation treatment during the same breeding season may negatively influence the consecutive recovery of oocytes. Proper rest is required for the animal after surgical oocyte removal (16). Another primary problem contributing to a lower number of recovered embryos is the production of high anti-eCG antibodies due to repeated administration of eCG hormones (32). Increasing the interval between repeated recoveries from the same animal and improving embryo recovery methods (16) could improve the potential for surgically recovering more embryos.

### 4.4. Superovulation and SCNT

Embryos produced through *in vivo* fertilization generally exhibit greater developmental competence regarding blastocyst formation rate compared to embryos obtained through *in vitro* fertilization (IVF) and SCNT-derived embryos. This may be because the oocytes used for IVF or SCNT are mostly obtained from an abattoir source, undergo *in vitro* maturation (IVM), have a relatively hyperoxic environment, and tend to have high oxidative stress levels. Furthermore, the overproduction of ROS induces apoptotic cell death, thereby impairing the quality and developmental potential of oocytes (88, 89). However, these situations do not occur *in vivo* cultures. Defects in an abnormal epigenetic status have been reported for SCNT-derived embryos due to inadequate remodeling of the donor nucleus.

Enucleated metaphase II oocytes, as recipient cytoplasm (90), are selected from the oocytes. Choosing to collect oocytes from slaughterhouse ovaries is a more convenient and cost-effective option compared to inducing ovulation in donor animals through multiple exogenous gonadotrophin hormone injections (91). Moreover, due to the low efficiency of the SCNT procedure, a relatively large number of oocytes are required to produce live offspring or conduct other meaningful experiments. While selecting oocytes from slaughterhouse ovaries may be easier and cost-effective, there is a lack of proper record-keeping regarding the animals' reproductive performance, age, management practices, and genetic origin.

There was no difference in the number of transferable embryos obtained from the cytoplasts of FSH-stimulated ovaries and embryos from the fusion of cytoplasts from abattoir ovaries (91). In sheep, a higher number of quality oocytes, pregnancy rates, and live kid rates were observed when using reconstructed embryos obtained from enucleated recipient oocytes obtained through FSH treatment compared to using ovaries from slaughterhouses (90). FSH pre-treatment improved oxygen consumption and OCT4 and IFN-τ expression in SCNT embryos, which indicates that FSH has a positive effect on oocyte quality (92). Therefore, it has become important to identify a noninvasive and non-disruptive method for selecting oocytes before culture (93). Oocytes obtained through superovulation may have a key role in obtaining better efficiency for nuclear transfer. In cattle and goats, it has been observed that *in vitro*-matured oocytes result in high prenatal and postnatal losses, poor embryo developmental competence, and lower pregnancy rates compared to *in vitro*-matured oocytes (91, 94–97). It was found that the oviductal oocytes (*in vivo*-matured oocytes) have a greater electrical pulse than the follicular oocytes (*in vitro*-matured oocytes). One of the 17 recipients delivered a normal live birth, and two pregnancies were achieved by transferring *in vivo*-matured embryos. On the other hand, no live births were obtained from *in vitro*-matured oocytes. Similarly, Martins et al. (98) also observed that no SCNT pregnancies reached term with the use of *in vitro-*matured oocytes, whereas *in vivo*-matured oocytes resulted in the successful birth of two transgenic cloned kids.

Reggio et al. (91) conducted a study comparing the *in vitro* developmental potential of nuclear transfer embryos produced by fusing transgenic donor cells with cytoplasts derived from the ovaries of animals that had undergone superovulation induced through multiple injections of FSH and abattoir ovaries. The results showed that the rate of fusion of NT embryos reconstructed from oocytes from either FSH-stimulated or abattoir-derived ovaries was 63 and 57%, respectively, which shows that oocyte source had no effect on embryo development or the overall pregnancy rate. However, the performance of oocytes selected using these vague criteria is often problematic and inaccurate, making it difficult to distinguish oocytes with different levels of developmental competence (93). Another study conducted by Peura et al. (99) reported no significant difference in blastocyst development rates (40.4 and 35.8%) between the high and low diet groups, but there was a significant difference in the established pregnancies (50 and 28.6%), resulting in live births. Multiple studies have shown that, after reprogramming by oocytes, SCNT embryos exhibit distinct gene expression patterns compared to *in vivo*-derived or i*n vitro*-fertilized (IVF) embryos (100). In sheep, SCNT embryos have been reported to exhibit slightly higher overall methylation levels than IVF embryos. However, the donor cell chromatin showed a conserved distribution when the transferred donor cell nuclei were compared to IVF embryonic nuclei (101).

## 5. The consequences of superovulation for oocytes and embryos

The superovulation process yields a large number of oocytes. However, their maturation rate can be hampered. Despite this barrier, it was found that the potential of mature oocytes to be fertilized and to develop into blastocysts is not affected by their origin (102). Despite substantial advancements in assisted reproduction technologies in recent years, the pregnancy rate remained low since a large proportion of transferred embryos fail to implant (103). Exogenous gonadotropin treatment leads to greater concentrations of circulating steroids, which may influence either oocyte or embryo quality, as well as the oviductal and/or uterine environment. This can also disrupt the synchronization that typically occurs between the embryo and the endometrium during the implantation process. Hence, a link may exist between the use of gonadotrophins for ovarian stimulation and the observed low implantation rate and gestational problems (104).

Several studies have shown that the yield of embryos produced through the induction of superovulation can be highly variable and negatively influenced by factors such as the effects on the oocyte during follicular growth or directly during embryo development in the oviduct and/or uterus (105, 106). The mechanism by which oocytes and embryos develop under hormonal superstimulation has an effect on abnormal endocrine conditions compared to those developed in unstimulated animals, ultimately leading to low developmental potential (107). Ovarian stimulation, or “superovulation,” induced by exogenous hormones may stimulate follicular development and oocyte maturation, resulting in the production of a greater number of oocytes. However, exogenous hormones affect the natural hormone environment, which is necessary for female reproduction, particularly follicle development and oocyte maturation (108). Superovulation may decrease the number of fertilized oocytes and preimplantation competence *in vivo*. The lower fertilization rate of oocytes obtained *in vivo* using superovulation methods may be attributed to sperm or oocyte transportation disturbances in the oviduct (109), alteration in the oviductal function such as the presence of carbohydrate residues on the epithelium of the ampulla (110), and the expression of specific genes (111). The variation in the timing of oocyte maturation between follicles during ovulation could also potentially lead to a reduced fertilization rate (112). The use of superovulation in ewes has been shown to reduce the number of sperm present in the oviduct (109). Previous studies on mice have shown that the COH procedures can cause delayed embryonic development, decreased implantation, and higher post-implantation loss (104). Another study reported that stimulated mouse embryos had delayed blastocyst formation, increased incidence of zonal lysis, and blastocyst collapse compared to naturally cycling controls (113).

Some studies suggest that superovulation can cause alterations in oocyte properties, and the *in vitro* fertilization rate in cows may be adjusted by altering the timing of FSH administration relative to oocyte harvesting (114). Thus, superovulation is likely to result in the ovulation of abnormal oocytes in at least some instances (109). Studies on mice have demonstrated the detrimental impact of ovarian stimulation on oocyte/embryo developmental competence, where the transfer of embryos from superovulated donors resulted in a considerably lower implantation rate in the control recipients than when embryos from control donors were transferred (104). Implications for the embryo as shown in mice, embryos produced from oocytes retrieved during superovulation may have reduced competence for preimplantation development *in vivo* (104, 113) in mice and cows (115). The oviduct and its fluid provide favorable conditions for gamete maturation, gamete transit, fertilization, and early embryonic development, which are crucial for mammalian reproduction and are regulated by steroids (115, 116).

These results showed that embryonic development *in vivo* before the transfer and superovulation was associated with reduced embryo competence for establishing pregnancy in recipients (113), increased fetal loss rates after the establishment of pregnancy (104), placental dysfunction (117), and reductions in fetal weight (117). The effects of superovulation may be influenced by the specific technique used to induce it, as well as other factors such as animal strain. Superovulation has no deleterious influence on embryonic and fetal development in pigs (118). Disruption of the development of embryos from superovulated females could have effects on the oocyte or alterations in the function of the oviduct and endometrium due to high concentrations of ovarian steroids in the blood of superovulated females.

Several studies on mammalian oocytes and embryos have shown that superovulation results in aberrant gene expression, including genes that are believed to be important for oocyte quality, cell cycle regulation, and inhibition (119). The gene expression patterns during blastocytes were found to be different in the embryos derived from the superovulated females. Ovarian stimulation triggers a cascade of hormonal and physiological events that create a different environment for oocyte maturation compared to naturally matured oocytes. This may also result in variations in the timing of ovulation. In addition, endometrial gene expression was altered by superovulation in both cows and humans (104). Embryos that are produced *in vivo* and those produced *in vitro* also exhibit differences in their gene expression and patterns of DNA methylation (120, 121). Therefore, the use of exogenous hormone stimulation can result in epigenetic changes in both oocytes and developing embryos. To understand the consequences of these changes during development, it is important to conduct controlled experiments that can dissect the epigenetic alterations that occur.

## 6. Conclusion

Successful studies have been conducted on simplified superovulation protocols using a combination of FSH and eCG in cattle and sheep. However, there were insufficient data on the successful simplification of superovulatory protocols with satisfactory ovarian responses in goats. Unlike cattle, further studies on simplification protocols based on the endocrinology profile of goats may not only reduce the cost of hormones but also make this technology more applicable to goats. Studies on immunization against inhibin along conventional superovulation protocols have shown successful results in cattle; however, there are limited data available, indicating a need for further studies to achieve a maximum ovarian response. The synchronization of the first follicular wave using the day-0 protocol showed a satisfactory response in terms of transferable embryos.

However, further studies are required to simplify this protocol by reducing the number of FSH injections from multiple injections to a single injection. A significant number of embryos undergo deterioration, and pregnancy rates remain low because a larger number of transplanted embryos fail to implant. In addition, a validation technique is required to determine whether candidate genes and putative SNP markers may contribute to oocyte quality, cell cycle regulation, inhibition, or a higher concentration of circulating hormones that can compromise the quality of embryos in the oviductal and even uterine environment, resulting in a reduced competency of embryos to establish a pregnancy.

## Author contributions

Conceptualization: SK, MJ, and YL. Software: IK. Validation: YL and SK. Resources, supervision, and funding acquisition: YL. Writing—original draft preparation: SK and MJ. Writing—review and editing: IK, AJ, and IU. Visualization: NK. Project administration: YL and IK. All authors have read and agreed to the published version of the manuscript.
